# Use of Healthcare Claims Data to Generate Real-World Evidence on Patients With Drug-Resistant Epilepsy: Practical Considerations for Research

**DOI:** 10.36469/001c.91991

**Published:** 2024-02-27

**Authors:** Nicole Stamas, Tom Vincent, Kathryn Evans, Qian Li, Vanessa Danielson, Reginald Lassagne, Ariel Berger

**Affiliations:** 1 Evidera, Bethesda, Maryland, USA; 2 LivaNova, London, UK

**Keywords:** administrative claims, methods, clinical coding, epilepsy, drug-resistant epilepsy, vagus nerve stimulation, neuromodulation

## Abstract

**Objectives:** Regulatory bodies, health technology assessment agencies, payers, physicians, and other decision-makers increasingly recognize the importance of real-world evidence (RWE) to provide important and relevant insights on treatment patterns, burden/cost of illness, product safety, and long-term and comparative effectiveness. However, RWE generation requires a careful approach to ensure rigorous analysis and interpretation. There are limited examples of comprehensive methodology for the generation of RWE on patients who have undergone neuromodulation for drug-resistant epilepsy (DRE). This is likely due, at least in part, to the many challenges inherent in using real-world data to define DRE, neuromodulation (including type implanted), and related outcomes of interest. We sought to provide recommendations to enable generation of robust RWE that can increase knowledge of “real-world” patients with DRE and help inform the difficult decisions regarding treatment choices and reimbursement for this particularly vulnerable population.

**Methods:** We drew upon our collective decades of experience in RWE generation and relevant disciplines (epidemiology, health economics, and biostatistics) to describe challenges inherent to this therapeutic area and to provide potential solutions thereto within healthcare claims databases. Several examples were provided from our experiences in DRE to further illustrate our recommendations for generation of robust RWE in this therapeutic area.

**Results:** Our recommendations focus on considerations for the selection of an appropriate data source, development of a study timeline, exposure allotment (specifically, neuromodulation implantation for patients with DRE), and ascertainment of relevant outcomes.

**Conclusions:** The need for RWE to inform healthcare decisions has never been greater and continues to grow in importance to regulators, payers, physicians, and other key stakeholders. However, as real-world data sources used to generate RWE are typically generated for reasons other than research, rigorous methodology is required to minimize bias and fully unlock their value.

## BACKGROUND

The US Food and Drug Administration (FDA) defines real-world data (RWD) as “data relating to patient health status and/or delivery of healthcare routinely collected from a variety of sources.”^1^ RWD include electronic medical records (EMR), administrative claims and billing data, product and disease registries, surveys, social media, mobile devices, and wearables. The FDA describes real-world evidence (RWE) as information obtained from analyses of RWD that can inform clinical practice, healthcare policy, and manufacturers’ strategies.^1^ Regulatory agencies from several countries now recognize the usefulness of RWE,^1–5^ as do health technology assessment (HTA) agencies, payers, physicians, and other key decision makers.^6–9^ While the increasing importance of RWD is clear, the challenges associated with its use to generate robust and rigorous RWE are less well understood.

For many reasons, epilepsy is an ideal condition to demonstrate challenges inherent in RWE generation. Epilepsy is a heterogeneous condition, with each type (eg, generalized, focal) characterized with specific diagnosis codes, although clinicians and billing specialists may differ on the diagnoses they select. Epilepsy has multiple stages and severity levels based on type(s) experienced and patient response to treatment, none of which are directly captured in secondary databases.^10^ It also is a prevalent condition, affecting approximately 2.3 million adults and 450,000 children in the United States^11^; roughly one-third of adults and one-quarter of children with epilepsy have drug-resistant epilepsy (DRE),^12^ which is defined by the International League Against Epilepsy as “failure of adequate trials of two tolerated and appropriately chosen anti-seizure medication (ASM) schedules (whether as monotherapies or in combination) to achieve sustained seizure freedom.”^13^ Neuromodulation (NM)–based interventions represent an evolving treatment alternative for patients with DRE. Three devices have been approved by the US FDA for patients with partial-onset seizures refractory to treatment by ASMs: vagus nerve stimulation (VNS), responsive neurostimulation (RNS), and deep brain stimulation (DBS).^14–16^ Within RWD, NM modalities are difficult to differentiate,^12^ as relevant procedure codes have varying levels of sensitivity and specificity. Important epilepsy-related variables, including seizure frequency and severity, are not recorded consistently or comprehensively, nor are decision-making processes (patient or clinician) related to therapy choice, severity of adverse events, impacts to family members and caregivers (especially in relation to indirect costs), or measures related to quality of life. Several algorithms have been developed to identify patients with epilepsy using data available in healthcare claims, although these still include biases due to the limitations of RWD.^10,17–21^

We identify and describe some challenges associated with RWE generation in epilepsy based on healthcare claims and offer potential solutions with illustrative examples based on our recent experience focused on patients who received NM for DRE.^22–24^ Our goal was to provide researchers with a holistic view of designing an RWE generation study (eg, allocation of exposure[s] and assessment of outcomes), specific to patients who underwent NM for DRE, using healthcare claims data.

## METHODOLOGIC RECOMMENDATIONS

### Data Source

Several sources of RWD exist—including claims, electronic medical records (EMR), social media data, registries, and survey data. The researcher should select the source that will enable examination of the research questions of interest. Healthcare claims data are a common RWD source from which RWE is generated because: (1) they are relatively straightforward to analyze; (2) they are relatively inexpensive to acquire (at least compared with other options); and (3) they provide a comprehensive view of all care received (and medications dispensed) for which insurance reimbursement is obtained, thereby enabling assessments of a broad number of outcomes relevant to payers—a key stakeholder in discussions related to the ability of patients to access appropriate care.

Healthcare claims databases comprise administrative claims that are generated by healthcare providers and adjudicated by insurers, along with enrollment data, including dates of eligibility for medical and pharmacy insurance coverage. Their primary purpose is to facilitate billing and reimbursement for healthcare services rendered and medications and durable medical equipment dispensed. Claims are ideal to assess patterns of use and cost of healthcare services and pharmacotherapies because they capture all care for which insurers provide reimbursement, including but not limited to information on services rendered (eg, procedures, diagnostics, administered therapies, medications dispensed by pharmacies) and amounts reimbursed by insurer and patient. However, claims lack reasons for treatment selection (or even if a treatment was discussed/suggested but ultimately rejected), results of diagnostic testing, vital statistics and other anthropomorphic data, and symptomatology; they also lack information on non-reimbursed services, including use of over-the-counter therapies. When selecting a data source—claims or other—we recommend first assessing its ability to support the planned study across a number of criteria. For example, the data source should include a sufficient sample size of patients with the particular indication and/or treatment of interest. The outcomes and exposures of the study—or reasonable proxies if necessary—must also be reliably measurable within the data set. Other considerations include the cost of the data set, the time period over which data are required, the time to access the data, and whether the data can be extracted and sent to the researcher for analysis (in some instances, data holders do not allow their data to be extracted and instead either require researchers to work virtually in a secure data environment or to submit their protocol and statistical analysis plan to the data holder, who then conducts the analysis).

In our study of patients who underwent NM for DRE, we used the Merative (formerly IBM) MarketScan® Commercial Database because it (1) captured relevant care across all healthcare providers who treat patients with DRE (ie, optimal comprehensiveness, given that DRE-related care is typically rendered in multiple settings); (2) included eligibility data, which allowed for the establishment of “time at risk” (ie, an enrolled day without claims can be assumed to indicate a day without utilization); and (3) included what is paid by insurers and patients for care (an important outcome in our study) (see **Study Timeline** below for more information on this topic). While US claims databases are broadly similar in terms of their composition and what is and is not available to researchers, the Merative database is relatively large, including longitudinal patient-level data for more than 273 million patients,^25^ including 28,218 patients who were deemed to have received a NM device within the time period of interest.

### Study Timeline

**Figure 1.**
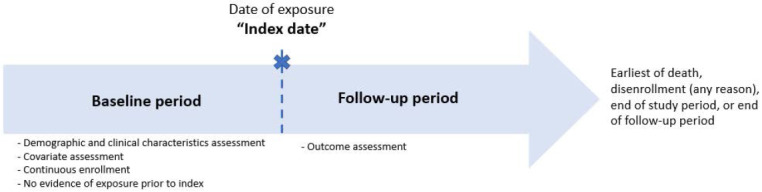
Study Timeline

When conducting comparative effectiveness assessments using secondary data, an important initial step is defining periods of interest. This may include the period over which relevant exposures (such as NM implantation) will be identified, relevant outcomes will be assessed, and important covariates established. Defining these periods allow the researcher to establish appropriate temporal relationships (ie, the relevant exposure[s] should occur before follow-up can begin; similarly, covariates and “baseline” characteristics of the sample should be established prior to receipt of exposure[s]) (**Figure 1**). The exposure date, or index date, reflects the date of initial diagnosis or initiation/receipt of a relevant treatment/procedure such as NM implantation; it also serves to demarcate patient time, with follow-up beginning on or after this date, and patient characteristics/covariates typically described using information available before this date.

Enrollment data, which are available in many claims databases, are important to establish “at risk” time, as a day enrolled where no utilization occurs can be considered a day without receipt of healthcare; conversely, as the database is developed from the perspective of health insurers who must reimburse care rendered on their members, no information on healthcare utilization is available for a day where the patient is not enrolled in the plan (for any reason). Compounding this problem is the fact that in the US, gaps in healthcare coverage due to changes in employment and/or insurance provider are frequent, resulting in periods of incomplete data within healthcare claims. Changing insurers is also relatively common—up to 22% of patients enrolled in US commercial (ie, private) health plans disenroll annually.^26^ Accordingly, one must balance the duration of a baseline period (with a continuous enrollment requirement to ensure maximal capture of relevant data) with the need to have sufficient patients for analysis. Depending on the data source and research question(s), the researcher may want to allow some maximum gap in coverage (eg, ≤14 days) to maximize the study sample while minimizing the likelihood of missing data. The US Centers for Medicare & Medicaid Services define a “short gap” in healthcare coverage as fewer than 3 consecutive months during the year without insurance coverage^27^; however, any allowed gaps introduce the possibility of missing data. We recommend conducting a preliminary assessment of the data to determine enrollment durations among patients, and to use that information (including the impact of continuous enrollment on potential sample size), coupled with what is known about the disease area and the research question(s) of interest, to inform decisions on whether gaps in coverage should be allowed and if so, how to define the maximum duration and number of such gaps.

However, there are several databases (including some claims databases) that do not include enrollment data. In such databases, researchers may impose a utilization-based “active” patient criterion in which patients are required to have a healthcare encounter documented within some time period prior to exposure assessment. The implicit assumption is that this patient would likely return to the same place for care, and therefore utilization serves as a proxy for active enrollment. However, the more stringent the utilization-based requirement imposed, the less generalizable the sample population becomes to the overall population of interest. Unfortunately, without additional (and likely time-consuming and expensive) validation efforts, there is no way to know either the amount of missing data that may fall into “gap days” or the degree to which any “active” patient criteria minimize the risk of missing data.

In our study, we defined the study period as spanning January 1, 2012, through December 31, 2019, which represented the most recent period for which data were available at the time the study was initiated. We identified all patients who underwent NM implantation (the exposure of interest) during this period. The earliest date on which NM implantation was identified was designated the index date. The 2-year period prior to the index date was designated the baseline period. Because many years may elapse between initial implantation and the need for device change/repair, we opted for a 2-year baseline period in our study and excluded all patients with any evidence of NM during baseline (ie, we believed some indicator of prevalent NM would be identifiable over a 2-year period but potentially not with a shorter period). Despite the negative impact on overall sample size—the required 2 years of continuous enrollment prior to the index date resulted in the loss of 58% of patients who underwent NM implantation—the relatively long baseline period maximized the likelihood that the identified index date represented incident NM implantation. Further, the relatively long baseline period also allowed for a more comprehensive capture of patient demographic and clinical characteristics, along with patterns of use and cost of healthcare services and pharmacotherapy prior to NM implantation. This helped shed light on overall patient burden prior to NM implantation, which in and of itself has increased knowledge on this particularly vulnerable population.^28^ Patients were required to be continuously enrolled over the entire 2-year baseline period (no gaps in enrollment were allowed to ensure complete data capture for selected patients). Patients were followed for a maximum of 2 years following NM implantation, or until death, disenrollment (any reason), or end of study database (whichever occurred first).

### Ascertainment of Exposures and Outcomes

**Exposure assessment:** Constitution of study measures should be informed by the type of RWD source(s) used, along with prior research (within the same or a similar source) where possible. We encourage a review of existing publications to inform indication-related operational definitions, including identification of methodologies that have been validated and/or implemented within the same RWD source (or type of RWD source) used for the study in question.^14,29–32^ Where possible, operational definitions should be reviewed by relevant experts to further ensure accuracy and appropriateness. In the context of RWE, “experts” refers to clinicians experienced in the relevant therapeutic area, as well as coding specialists (where applicable). To mitigate issues related to misclassification, researchers may require multiple instances of relevant codes on different days; they may also consider use of procedures, medications, and/or visit type as further evidence (our study required codes for NM, epilepsy, and ASMs—all of which are expected in DRE).^33–35^ Prior work has found that definitions that incorporate diagnoses and medications (at least medications specific to the disease in question) perform better than those limited to diagnoses alone.^20^ If needed, proxies should be used when relevant information is unavailable; as relevant, operational definitions should account for coding practices and/or differences in available codes over time. Should detailed clinical data be available, we encourage its use to further increase specificity of case definitions and reduce risk of misclassification. Definitions may include information that can illustrate disease severity (eg, hospitalizations, with corresponding relevant diagnosis codes, or even total healthcare costs [when available] as a proxy for overall levels of morbidity), antecedent therapies (eg, numbers and types of prescription dispenses, potentially establishing lines of therapy), and other disease-specific aspects. We also recommend performing sensitivity analyses where possible, to allow for more insight into possible variations in the estimates for outcome measures, especially for estimates that involve proxy measures or variations on case definitions.

For measures related to devices, such as NM devices, we recommend reviewing manufacturers’ billing guidance, as these presumably are used by providers to minimize the likelihood of provider underpayment due to erroneous or incomplete coding; we also recommend verifying that multiple modalities are not identified on the same day (ie, coding inconsistencies), which we identified in a minority of patients in our sample (RWD are not always collected with the same rigor as data collected during the course of a clinical study). While the decision to include or exclude any particular patient is left to the researcher, we recommend removing patients for whom exposure cannot be definitively ascertained.

It is also important to note that coding inconsistencies are frequent within claims data, and that data not required to support reimbursement are not always entered with the same care as those that do. This is because claims information is used for billing purposes rather than clinical or research purposes. Accordingly, requiring multiple evidentiary points to inform sample selection minimizes the impact of any such inconsistency or error.

As mentioned above, in our study we excluded all patients without continuous enrollment over the baseline period to ensure complete capture of all relevant information related to NM, epilepsy, and important covariates. Because our goal was to focus on a DRE cohort undergoing initial NM device implantation, we dropped all patients with evidence of (1) NM at any time during the baseline period (eg, procedure codes for neurostimulator removal, replacement, repair, analysis, programming, or complication); (2) multiple devices (ie, VNS, RNS, DBS) implanted on the index date; or (3) evidence of cranial epilepsy surgery (an alternative surgical intervention for DRE) at any time during baseline. We reviewed relevant product manuals^36–38^ and published studies^22–24^ to define NM-related codes (implantation, revision, repair, device [re]programming, battery repair) (**Table 1**). As shown in **Table 1**, some billing codes were specific to individual devices; some were insufficiently specific to differentiate between NM products; some covered implantation of any NM device; others could differentiate between implantation sites (eg, chest, cranium/skull); and others were insufficiently specific to identify insertion site or NM type. We decided to only use specific surgical codes that occurred prior to any other NM codes (eg, revision, programming) to identify the first date of implantation.

**Table 1.** All NM-Related Procedure Codes Identified Among Patients^a^ During the Study Period, by Code Specificity and Billing Categorization

**Code Type**	**Code**	**Code Description**	**Specificity**	**Categorization**
CPT	64568	Incision for implantation of cranial nerve (eg, vagus nerve) neurostimulator electrode array and pulse generator	Specific	VNS
CPT	64569	Revision or replacement of cranial nerve (eg, vagus nerve) neurostimulator electrode array, including connection to existing pulse generator	Specific	VNS
CPT	64570	Removal of cranial nerve (eg, vagus nerve) neurostimulator electrode array and pulse generator	Specific	VNS
CPT	95976	Electronic analysis of implanted neurostimulator pulse generator/transmitter (eg, contact group[s], interleaving, amplitude, pulse width, frequency [Hz], on/off cycling, burst, magnet mode, dose lockout, patient selectable parameters, responsive neuromodulation, detection algorithms, closed loop parameters, and passive parameters) by physician or other qualified healthcare professional; with simple cranial nerve neurostimulator pulse generator/transmitter programming by physician or other qualified healthcare professional	Specific	VNS
CPT	95977	Electronic analysis of implanted neurostimulator pulse generator/transmitter (eg, contact group[s], interleaving, amplitude, pulse width, frequency [Hz], on/off cycling, burst, magnet mode, dose lockout, patient selectable parameters, responsive neuromodulation, detection algorithms, closed loop parameters, and passive parameters) by physician or other qualified healthcare professional; with complex cranial nerve neurostimulator pulse generator/transmitter programming by physician or other qualified healthcare professional	Specific	VNS
CPT	61867	Twist drill, burr hole, craniotomy, or craniectomy with stereotactic implantation of neurostimulator electrode array in subcortical site (eg, thalamus, globus pallidus, subthalamic nucleus, periventricular, periaqueductal gray), with use of intraoperative microelectrode recording; first array	Specific	DBS
CPT	61868	Twist drill, burr hole, craniotomy, or craniectomy with stereotactic implantation of neurostimulator electrode array in subcortical site (eg, thalamus, globus pallidus, subthalamic nucleus, periventricular, periaqueductal gray), with use of intraoperative microelectrode recording; each additional array (list separately in addition to primary procedure)	Specific	DBS
CPT	64999	Cranial implantation or replacement of neurostimulator pulse generator	Specific	RNS
CPT	61850	Craniectomy or craniotomy for implantation of neurostimulator electrodes, cerebral, cortical	Specific	RNS
CPT	61860	Electrocorticogram from an implanted brain neurostimulator pulse generator/transmitter, including recording, with interpretation and written report, up to 30 days	Specific	RNS
CPT	95836	Insertion of neurostimulator lead into brain, percutaneous endoscopic approach	Specific	RNS
CPT	61863	Twist drill, burr hole, craniotomy, or craniectomy with stereotactic implantation of neurostimulator electrode array in subcortical site (eg, thalamus, globus pallidus, subthalamic nucleus, periventricular, periaqueductal gray), without use of intraoperative microelectrode recording; first array	Nonspecific	RNS/DBS
CPT	61864	Twist drill, burr hole, craniotomy, or craniectomy with stereotactic implantation of neurostimulator electrode array in subcortical site (eg, thalamus, globus pallidus, subthalamic nucleus, periventricular, periaqueductal gray), without use of intraoperative microelectrode recording; each additional array (list separately in addition to primary procedure)	Nonspecific	RNS/DBS
CPT	61880	Revision or removal of intracranial neurostimulator electrodes	Nonspecific	RNS/DBS
CPT	61885	Insertion or replacement of cranial neurostimulator pulse generator or receiver, direct or inductive coupling; with connection to a single electrode array	Nonspecific	RNS/DBS/VNS
CPT	61886	Insertion or replacement of cranial neurostimulator pulse generator or receiver, direct or inductive coupling; with connection to 2 or more electrode arrays	Nonspecific	RNS/DBS
CPT	95983	Electronic analysis of implanted neurostimulator pulse generator/transmitter (eg, contact group[s], interleaving, amplitude, pulse width, frequency [Hz], on/off cycling, burst, magnet mode, dose lockout, patient selectable parameters, responsive neuromodulation, detection algorithms, closed loop parameters, and passive parameters) by physician or other qualified healthcare professional; with brain neurostimulator pulse generator/transmitter programming, first 15 minutes face-to-face time with physician or other qualified healthcare professional	Nonspecific	RNS/DBS
CPT	95984	Electronic analysis of implanted neurostimulator pulse generator/transmitter (eg, contact group[s], interleaving, amplitude, pulse width, frequency [Hz], on/off cycling, burst, magnet mode, dose lockout, patient selectable parameters, responsive neuromodulation, detection algorithms, closed loop parameters, and passive parameters) by physician or other qualified healthcare professional; with brain neurostimulator pulse generator/transmitter programming, each additional 15 minutes face-to-face time with physician or other qualified healthcare professional (list separately in addition to code for primary procedure)	Nonspecific	RNS/DBS
CPT	61888	Revision or removal of cranial neurostimulator pulse generator or receiver	Nonspecific	RNS/DBS/VNS
CPT	95970	Electronic analysis of implanted neurostimulator pulse generator/transmitter (eg, contact group[s], interleaving, amplitude, pulse width, frequency [Hz], on/off cycling, burst, magnet mode, dose lockout, patient selectable parameters, responsive neuromodulation, detection algorithms, closed loop parameters, and passive parameters) by physician or other qualified healthcare professional; with brain, cranial nerve, spinal cord, peripheral nerve, or sacral nerve, neurostimulator pulse generator/transmitter, without programming	Nonspecific	RNS/DBS/VNS
HCPCS	C1778	Lead, neurostimulator (implantable)	Specific	VNS
HCPCS	L8680	Implantable neurostimulator electrode, each	Nonspecific	VNS/RNS
HCPCS	C1787	Patient programmer, neurostimulator	Specific	DBS
HCPCS	C1820	Generator, neurostimulator (implantable), with rechargeable battery and charging system	Specific	DBS
HCPCS	C1883	Adapter/extension, pacing lead or neurostimulator lead (implantable)	Specific	DBS
HCPCS	L8679	Implantable neurostimulator, pulse generator, any type	Specific	DBS
HCPCS	L8681	Patient programmer (external) for use with implantable programmable neurostimulator pulse generator, rep	Specific	DBS
HCPCS	L8687	Implantable neurostimulator pulse generator, dual array, rechargeable, includes extension	Specific	DBS
HCPCS	C1767	Generator, neurostimulator (implantable), non-rechargeable	Nonspecific	RNS/DBS/VNS
HCPCS	L8688	Implantable neurostimulator pulse generator, dual array, non-rechargeable, includes extension	Nonspecific	RNS/DBS
HCPCS	L8686	Implantable neurostimulator pulse generator, single array, non-rechargeable, includes extension	Nonspecific	RNS/DBS/VNS
ICD-10	0JH60BZ	insertion of single array stimulator generator into chest subcutaneous tissue and fascia, open approach	Specific	DBS
ICD-10	0JH60DZ	Insertion of multiple array stimulator generator into chest subcutaneous tissue and fascia, open approach	Specific	DBS
ICD-10	0JH60EZ	Insertion of multiple array rechargeable stimulator generator into chest subcutaneous tissue and fascia	Specific	DBS
ICD-10	0JPT0MZ	Removal of stimulator generator from trunk subcutaneous tissue and fascia, open approach	Specific	DBS
ICD-10	0JPT3MZ	Removal of stimulator generator from trunk subcutaneous tissue and fascia, percutaneous approach	Specific	DBS
ICD-10	00P03MZ	Removal of neurostimulator lead from brain, percutaneous approach	Specific	DBS
ICD-10	00H04MZ	Insertion of neurostimulator generator into skull, open approach	Specific	RNS
ICD-10	0NH00NZ	Removal of neurostimulator generator from skull, open approach	Specific	RNS
ICD-10	0NP00NZ	Cranial implantation or replacement of neurostimulator pulse generator	Specific	RNS
ICD-10	00H00MZ	Insertion of neurostimulator lead into brain, open approach	Nonspecific	RNS/DBS
ICD-10	00H03MZ	Insertion of neurostimulator lead into brain, percutaneous approach	Nonspecific	RNS/DBS
ICD-10	00P00MZ	Removal of neurostimulator lead from brain, open approach	Nonspecific	RNS/DBS
ICD-9	8605	Incision with removal of foreign body or device from skin and subcutaneous tissue	Specific	DBS
ICD-9	8694	Insertion or replacement of single array neurostimulator pulse generator, not specified as rechargeable	Specific	DBS
ICD-9	8695	Insertion or replacement of multiple array neurostimulator pulse generator, not specified as rechargeable	Specific	DBS
ICD-9	8696	Insertion or replacement of other neurostimulator pulse generator	Specific	DBS
ICD-9	8697	Insertion or replacement of single array rechargeable neurostimulator pulse generator	Specific	DBS
ICD-9	8698	Insertion or replacement of multiple array (two or more) rechargeable neurostimulator pulse generator	Specific	DBS
ICD-9	0120	Removal of cranial neurostimulator pulse generator	Specific	RNS
ICD-9	0129	Twist drill or burr hole(s) for implantation of neurostimulator electrodes, cortical	Specific	RNS
ICD-9	0296	Insertion of sphenoidal electrodes	Nonspecific	RNS/DBS
ICD-9	0122	Removal of intracranial neurostimulator lead(s)	Nonspecific	RNS/DBS
ICD-9	0293	Implantation or replacement of intracranial neurostimulator lead(s)	Nonspecific	RNS/DBS

^a^Demonstrated codes are among all patients with any neuromodulation-related procedure code during the exposure identification period (January 1, 2012–December 31, 2019) and prior to implementation of additional exclusion criteria (eg, exclusion of codes indicating device removal/replacement).

Abbreviations: CPT, Current Procedural Terminology; DBS, deep brain stimulation; HCPCS, Healthcare Common Procedure Coding System; ICD-9, *International Classification of Diseases, Ninth Revision*; ICD-10, *International Classification of Diseases, Tenth Revision*; RNS, responsive neurostimulation; VNS, vagus nerve stimulation.

The researcher also should know that codes may change over time. For example, procedure codes specific to VNS changed in 2019, when CPT-4 codes 95974 and 95975 were removed, and CPT-4 codes 95976 and 95977 were added.^37^ Researchers should understand how treatments are billed over time, and develop operational definitions that ensure that these changes are considered, and are specific to the RWD source (including country), and year(s) of interest.

As noted above, 58% of patients who underwent NM implantation were excluded from our study due to insufficient durations of continuous enrollment during the 2-year baseline period. Consequently, establishing incident epilepsy, DRE (which cannot occur until sometime after the diagnosis of epilepsy has been established), and date of implantation (which may occur as late as 20 years following DRE onset,^39,40^ whereas mean duration of continuous enrollment in private [ie, commercial] US insurance plans is only 1.4 years^26^) was thought to be unfeasible within these data. Moreover, while there are diagnosis codes specific to “intractable” epilepsy, they are not consistently used. Accordingly, we started with the date of NM implantation based on procedure codes only, and then worked backward to ascertain: (1) the presence of epilepsy (diagnosis required to be identified on the date of implantation); and (2) one or more claims for ASMs during the 12 months prior to the implant date, with the assumption that clinicians would not offer NM until multiple ASM regimens had failed. Our definition was therefore based on knowledge of what was and was not available in the database, existing NM billing requirements,^36–38,41–46^ and previously utilized definitions in literature.^12,18^

While all patients in the sample had evidence of epilepsy on index date, that does not necessarily mean the procedure was performed for that condition. Consequently, with claims data, it was important to establish both the presence of the desired condition *and* the absence of other conditions for which the procedure could have been performed. In our study, this meant we sought to maximize the likelihood that NM was implanted for the management of DRE rather than another condition for which NM can be utilized, including essential tremors, incontinence, depression, and Parkinson’s disease.^14,29–32,47–49^ The reason each patient undergoes a particular procedure is not recorded in claims data; therefore, one must find alternative methods to ensure the procedure was not undertaken for other indications. For example, we discovered a number of patients with at least 1 diagnosis of epilepsy within 30 days of NM implantation but not on their index date: many of these patients had diagnoses for bladder control/incontinence (another condition for which NM can also be used^39^) on their index date; others had diagnoses of Parkinson’s disease. We ultimately opted to ensure a diagnosis of epilepsy was present on the index date, and to exclude all of these patients with “competing” explanations for NM to avoid risk of misclassification (**Table 2**).

**Table 2.** Case Study Attrition

**Selection Criterion**	**N**
≥1 claim resulting in procedure codes for VNS, RNS, and/or DBS between Jan. 1, 2012, and Dec. 31, 2019	28,218
Diagnosis of epilepsy on index date	4872
Outpatient pharmacy dispense of an ASM during 12-month period before and including index date	3824
Continuous enrollment for the 24-month period before and including index date	1600
No evidence of craniotomy or insertion or replacement of cranial neurostimulator pulse generator or receiver (RNS/DBS only), or of device removal/replacement (all 3 devices) during the 24-month period prior to index date. No evidence of multiple neurostimulators implanted on index date	876
No evidence of cranial epilepsy surgery or Parkinson’s disease during 24-month period before index date	860

Abbreviations: ASM, anti-seizure medication; CPT, Current Procedural Terminology; DBS, deep brain stimulation; RNS, responsive neurostimulation; VNS, vagus nerve stimulation.

While conditions like Parkinson’s disease enable relatively straightforward decisions, others, such as depression, which can be another reason to use NM or a consequence of DRE, do not. Approximately one-third of patients with DRE have been reported to have comorbid depression; excluding these patients would therefore risk reducing study generalizability.^24^ In studies of NM for DRE, we recommend including patients with comorbid depression and subsequently conducting stratified analyses to better understand how NM impacts those with and without this comorbidity.

### Outcomes Assessment

Claims data do not include information on seizure frequency or severity, likely because these events are a common occurrence (often occurring multiple times daily among persons with DRE) with which patients learn to live without seeking care upon the advent of each seizure. Patients often keep seizure diaries and record individual seizure events, along with specific triggers, which can be shared with providers at scheduled visits to assess effectiveness of epilepsy management and inform treatment changes.^39,50^ These diaries are not typically readily linkable to claims. Conversely, one benefit of healthcare claims data is its capture of robust economic and utilization data, including “costs.” While the term can represent what is paid for care, what is charged for care, or expenditures needed to render care, RWD sources tend to be limited to reimbursed amounts or charges, and healthcare claims represent total amounts paid (or charged). Depending on the research question(s), the researcher may wish to focus only on amounts paid by insurers or those paid by patients; regardless, researchers also should specify what their cost data represent. Because the study period often spans multiple years, the researcher may consider use of an inflation factor (eg, the medical care component of the Consumer Price Index) to allow all cost data to be presented using a common “reference” year.^51^

Our study therefore maximized information available in healthcare claims and assessed incidence of epilepsy-related hospital admissions, epilepsy-related emergency department visits, and epilepsy-related accidents, with the assumption that such services represented care likely rendered for severe seizures and/or other unwanted sequelae of epilepsy. As with NM (or exposure) ascertainment, operational definitions for outcomes should be constructed with sufficient sensitivity and specificity to exclude rule-out conditions and coding errors. In our study we defined epilepsy-related utilization and costs as all medical (ie, inpatient and outpatient) claims resulting in an epilepsy diagnosis code (any position) and all ASM dispenses. This broad definition is more sensitive as to not miss visits or costs that could be epilepsy-related. A sensitivity analyses was also conducted with a more specific definition requiring a primary diagnosis of epilepsy on medical claims (as well as all ASM dispenses) for utilization and costs to be considered epilepsy-related. The latter definition is more likely to only included epilepsy-related utilization and cost but may also underestimate the true utilization and cost associated with epilepsy.

### Conducting Comparative Effectiveness Assessments

Unlike randomized-controlled trials, exposures are not randomly assigned in studies based on RWD.^52^ The lack of randomization can result in selection bias and significant differences between exposed and unexposed patients that serve to confound analyses of the relationship between exposure and outcome (ie, differences in outcomes may be due to differences between groups other than exposure status).^53^ There are several methods to minimize risk of confounding, including but not limited to stratification and matching exposed patients to unexposed patients based on various potential confounding variables (ie, covariates). The latter approach can be achieved through propensity score matching; propensity scores also can be used to inform another means by which exposed and unexposed groups can be balanced called inverse probability of treatment weighting, in which weights are assigned to each patient based on their probability of exposure.^53^ Regardless of whether any of these methods are used, multivariable regression models can also be developed and used to provide (additional) adjustment for potential confounding. While it is beyond the scope of this paper to delve into these methods, researchers should employ appropriate methods that account for potential confounding that maximize available data and assess the degree to which these methods have resulted in

balanced treatment/exposure groups prior to conducting comparisons. The method to adjust for confounding should be considered carefully, as each has strengths and limitations that may be more or less relevant, depending on specific study question(s) and data source(s).

In our study, we used propensity score matching with a “greedy” nearest neighbor approach^54^ to match (1:1) a maximal number of VNS patients to their RNS/DBS counterparts; a specific radius (0.2 times the SD of the log-transformed propensity score distribution) was used for matching.^24^ Standardized differences were used to assess the balance of variables across the 2 cohorts following propensity score matching. One example of an important confounding variable in our study was age. While all NM devices (VNS and RNS, and DBS) are indicated for adults, only VNS is also indicated for pediatric patients.^14–16^ Therefore, pediatric patients are much more likely to have received VNS than other devices. Relatedly, patterns of use and cost of healthcare services may differ between pediatric and adult patients. Accordingly, it is important to ensure that age (along with other covariates) is balanced between VNS and RNS/DBS patients; we did so through incorporation of this measure into the model used to derive the propensity score. There are many other reasons physicians may choose one device over the other, including personal preference, experience (or lack thereof), where one practices, and insurance requirements/allowances. Many of these decisions cannot be measured directly in healthcare claims, and must instead be assessed through proxies. Among such proxies, we recommend inclusion of measures focused on use and cost of healthcare services (either all-cause or disease-specific, depending on the research question), as these parameters are good proxies for overall levels of morbidity and/or disease severity. **Table 3** illustrates the distribution of selected covariates in VNS vs RNS/DBS patients before and after propensity score matching.

**Table 3.** Distribution of Selected Patient Covariates Before and After Propensity Score Matching

**Measure**	**Before Matching**			**After Matching**		
	**VNS (N=640)**	**RNS/DBS (N=152)**	**Std. Diff.**	**VNS (N=148)**	**RNS/DBS (N=148)**	**Std. Diff.**
Age, mean (SD), y	25.2 (16.1)	32.1 (16.8)	0.50	32.8 (17.2)	31.7 (16.7)	0.06
Patients with epilepsy-related inpatient admission, n (%)	328 (51.3)	122 (80.3)	0.51	105 (70.9)	118 (79.7)	0.04
Patients with epilepsy-related ED visits, n (%)	367 (57.3)	72 (47.4)	-0.25	76 (51.4)	72 (48.6)	-0.10

Abbreviations: DBS, deep brain stimulation; ED, emergency department; RNS, responsive neurostimulation; SD, standard deviation; Std.Diff., standardized difference; VNS, vagus nerve stimulation.

## DISCUSSION

Regulatory agencies, payers, clinicians, and other decision-makers have historically prioritized randomized clinical trials to inform their decision making. Although the strict selection criteria and bespoke data collection activities of these trials maximize internal validity by limiting attribution of outcomes to the studied exposure(s), this is at the expense of generalizability.^55^ For example, one clinical trial limited to four selection criteria excluded 73% of eligible patients^56^; in another, only 4% to 7% of “real-world” individuals with the condition of interest met eligibility criteria from 30 published randomized clinical trials.^55^ Epilepsy trials that assess the efficacy of management strategies (eg, ASMs, NM) frequently exclude patients with chronic comorbidities and psychiatric conditions to evaluate treatment(s) in “laboratory conditions,” thus substantially reducing generalizability.^57^ Consequently, stakeholders have increasingly recognized the importance of RWE to inform healthcare practice and policy using information generated on relatively large and heterogenous populations treated in real-world settings.

However, working with RWD is challenging, not least because these data are often initially collected for reasons other than research. Consequently, researchers need to be diligent in their methodology. While choice of patient population(s) and research question(s) should drive the decision as to which RWD source(s) should be used, source-specific limitations and issues must subsequently be identified, investigated, mitigated, and described to yield reliable and reproducible observational research. Different RWD types have different strengths and weaknesses. Choice of RWD therefore should be informed by the research question(s), as that will inform population and outcomes of interest. In evaluating different RWD sources, one should also consider the ability to identify relevant exposure(s) and examine outcomes of interest, the likelihood of missing/incomplete data, and the potential impacts that these issues may have on sample selection and analyses. Variables used to assess exposures, outcomes, and covariates will be limited to those available within the selected RWD source; accordingly, researchers should also factor these concerns into their decision-making process.

The study timeline should be developed thoughtfully and provide for the ability to assign exposure temporally in relation to indication of interest, ascertain covariates, and assess outcomes; all decisions with respect to these and related issues also need to be balanced against concerns such as selection bias and immortal time bias. Constitution of study measures should be informed by the RWD source selected and prior research (within the same or a similar source) where possible. We encourage a review of existing publications to inform operational definitions, including where available methodologies have been validated and/or implemented within the same RWD source that was used for the study in question.^21,58–61^ Where possible, operational definitions should be reviewed by relevant experts to further ensure accuracy and appropriateness. If needed, proxies should be used when relevant information is unavailable; as relevant, operational definitions should account for coding practices and/or differences in available codes over time.

With the exception of pragmatic trials, RWE studies are not typically able to randomly allocate exposure and consequently present opportunities for residual and unmeasured confounding and other potential biases. While beyond our scope herein, analytic options to address these concerns include matching, weighting, stratification, and multivariable regression.^41–46^ Ultimately, to maximize internal and external validity of the study, the investigator should fully describe methods used and acknowledge any potential for residual confounding and bias, including its possible impact on study findings.

These recommendations apply to claims-based research on DRE; however, the general concepts described above are, in the collective opinion of the authors, relevant for a broader range of RWE generation studies.

## CONCLUSION

The importance of RWE continues to grow and inform all aspects of healthcare decision making, including but not limited to those related to decisions related to treatment and outcomes of DRE. While opportunities have never been greater, so too is the need for methodologically rigorous and robust approaches to maximize the ability to use RWE to improve patients’ health and quality of life.

### Disclosures

V.D. and R.L. are paid employees and potential shareholders of LivaNova, a manufacturer of the VNS neuromodulation device and other medical devices. K.E., Q.L., and A.B., are employees of Evidera, a health economics and outcomes research consultancy that received funding from LivaNova to conduct this research. At the time this research was conducted, N.S. and T.V. also were employees of Evidera.
